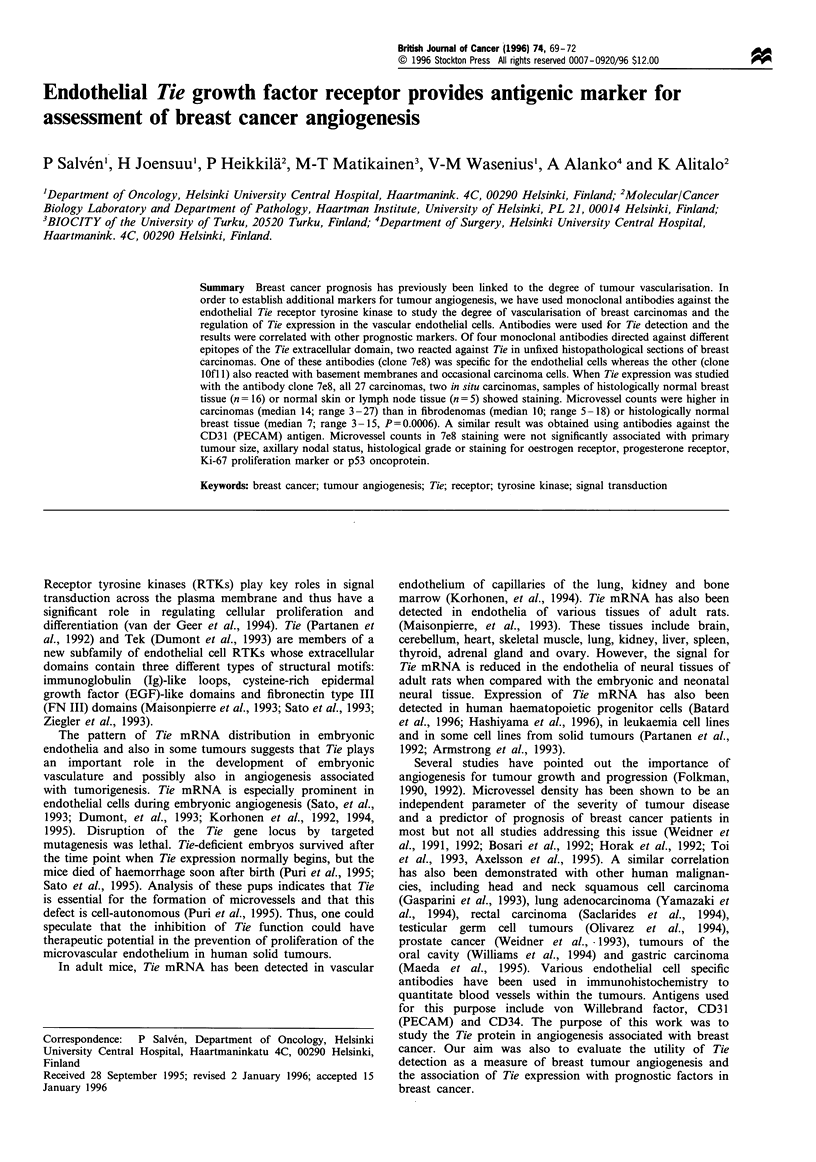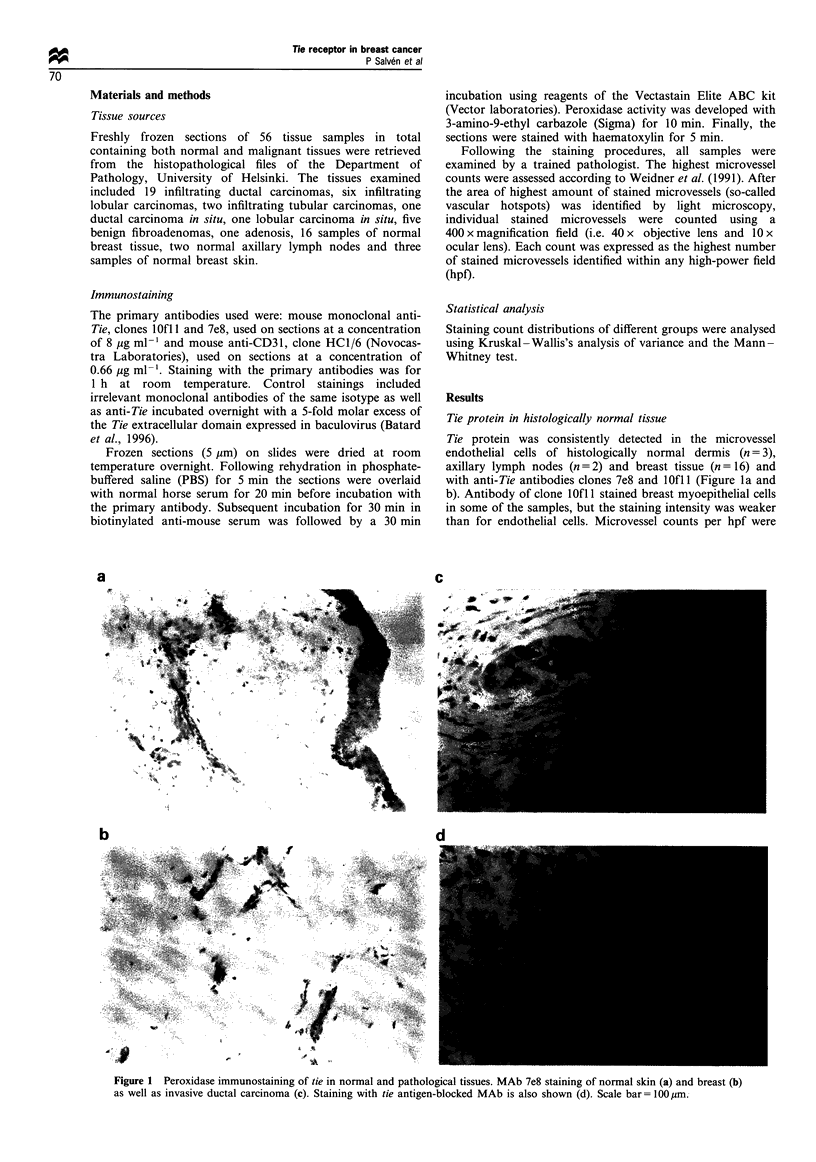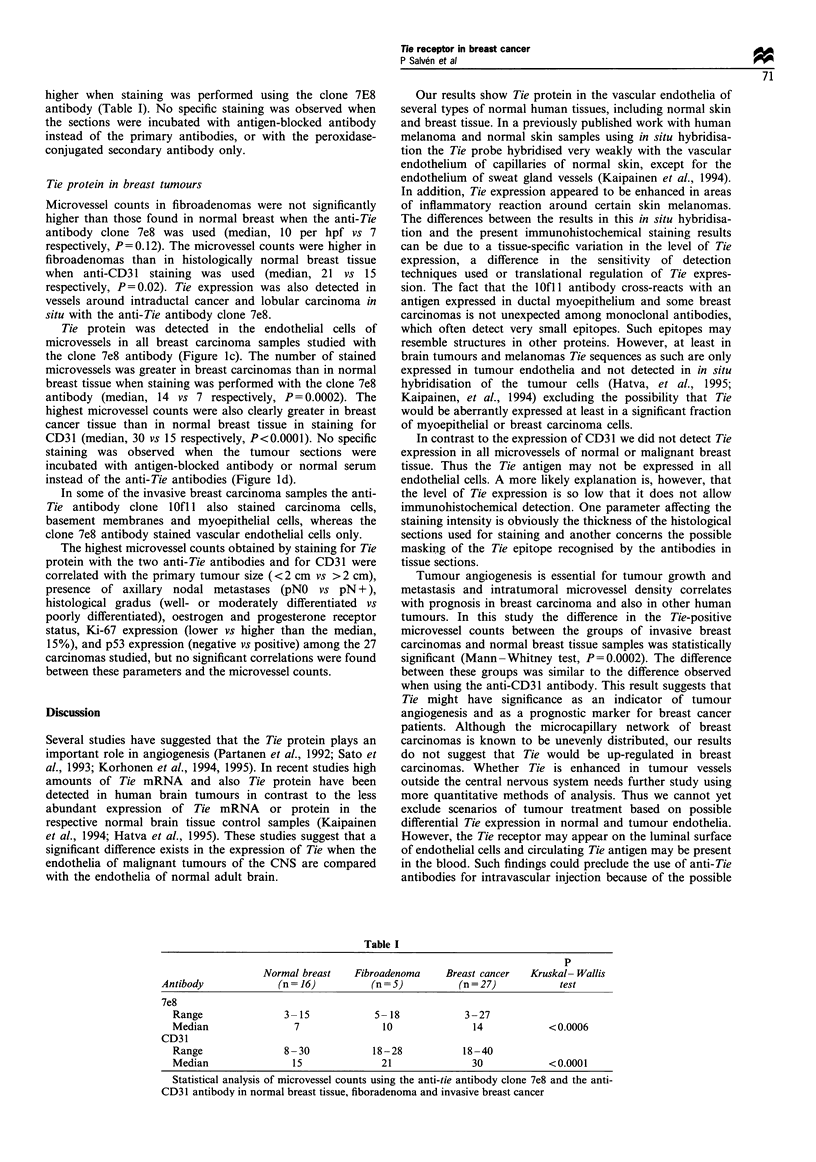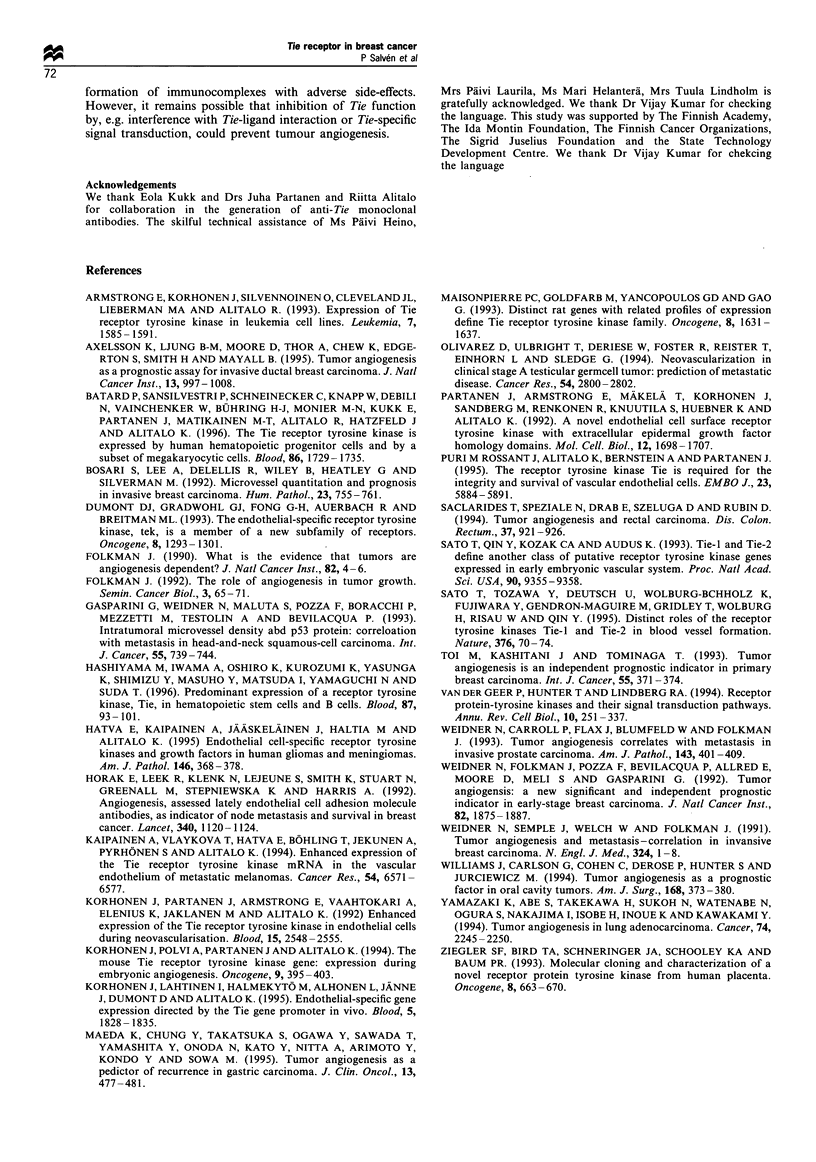# Endothelial Tie growth factor receptor provides antigenic marker for assessment of breast cancer angiogenesis.

**DOI:** 10.1038/bjc.1996.317

**Published:** 1996-07

**Authors:** P. Salvén, H. Joensuu, P. Heikkilä, M. T. Matikainen, V. M. Wasenius, A. Alanko, K. Alitalo

**Affiliations:** Department of Oncology, Helsinki University Central Hospital, Finland.

## Abstract

**Images:**


British Journal of Cancer (1996) 74, 69-72
© 1996 Stockton Press All rights reserved 0007-0920/96 $12.00

### Endothelial *Tie* growth factor receptor provides antigenic marker for assessment of breast cancer angiogenesis

P Salvén[1], H Joensuu[1], P Heikkilä[2], M-T Matikainen[3], V-M Wasenius[1], A Alanko[4] and K Alitalo[2]

*[1]Department of Oncology, Helsinki University Central Hospital, Haartmanink. 4C, 00290 Helsinki, Finland; [2]Molecular/Cancer Biology Laboratory and Department of Pathology, Haartman Institute, University of Helsinki, PL 21, 00014 Helsinki, Finland; [3]BIOCITY of the University of Turku, 20520 Turku, Finland; [4]Department of Surgery, Helsinki University Central Hospital, Haartmanink. 4C, 00290 Helsinki, Finland.*

**Summary** Breast cancer prognosis has previously been linked to the degree of tumour vascularisation. In order to establish additional markers for tumour angiogenesis, we have used monoclonal antibodies against the endothelial *Tie* receptor tyrosine kinase to study the degree of vascularisation of breast carcinomas and the regulation of *Tie* expression in the vascular endothelial cells. Antibodies were used for *Tie* detection and the results were correlated with other prognostic markers. Of four monoclonal antibodies directed against different epitopes of the *Tie* extracellular domain, two reacted against *Tie* in unfixed histopathological sections of breast carcinomas. One of these antibodies (clone 7e8) was specific for the endothelial cells whereas the other (clone 10f11) also reacted with basement membranes and occasional carcinoma cells. When *Tie* expression was studied with the antibody clone 7e8, all 27 carcinomas, two *in situ* carcinomas, samples of histologically normal breast tissue ($n=16$) or normal skin or lymph node tissue ($n=5$) showed staining. Microvessel counts were higher in carcinomas (median 14; range 3-27) than in fibrodenomas (median 10; range 5-18) or histologically normal breast tissue (median 7; range 3-15, $P=0.0006$). A similar result was obtained using antibodies against the CD31 (PECAM) antigen. Microvessel counts in 7e8 staining were not significantly associated with primary tumour size, axillary nodal status, histological grade or staining for oestrogen receptor, progesterone receptor, Ki-67 proliferation marker or p53 oncoprotein.

**Keywords:** breast cancer; tumour angiogenesis; *Tie*; receptor; tyrosine kinase; signal transduction

Receptor tyrosine kinases (RTKs) play key roles in signal transduction across the plasma membrane and thus have a significant role in regulating cellular proliferation and differentiation (van der Geer *et al.*, 1994). *Tie* (Partanen *et al.*, 1992) and Tek (Dumont *et al.*, 1993) are members of a new subfamily of endothelial cell RTKs whose extracellular domains contain three different types of structural motifs: immunoglobulin (Ig)-like loops, cysteine-rich epidermal growth factor (EGF)-like domains and fibronectin type III (FN III) domains (Maisonpierre *et al.*, 1993; Sato *et al.*, 1993; Ziegler *et al.*, 1993).

The pattern of *Tie* mRNA distribution in embryonic endothelia and also in some tumours suggests that *Tie* plays an important role in the development of embryonic vasculature and possibly also in angiogenesis associated with tumorigenesis. *Tie* mRNA is especially prominent in endothelial cells during embryonic angiogenesis (Sato, *et al.*, 1993; Dumont, *et al.*, 1993; Korhonen *et al.*, 1992, 1994, 1995). Disruption of the *Tie* gene locus by targeted mutagenesis was lethal. *Tie*-deficient embryos survived after the time point when *Tie* expression normally begins, but the mice died of haemorrhage soon after birth (Puri *et al.*, 1995; Sato *et al.*, 1995). Analysis of these pups indicates that *Tie* is essential for the formation of microvessels and that this defect is cell-autonomous (Puri *et al.*, 1995). Thus, one could speculate that the inhibition of *Tie* function could have therapeutic potential in the prevention of proliferation of the microvascular endothelium in human solid tumours.

In adult mice, *Tie* mRNA has been detected in vascular endothelium of capillaries of the lung, kidney and bone marrow (Korhonen, *et al.*, 1994). *Tie* mRNA has also been detected in endothelia of various tissues of adult rats. (Maisonpierre, *et al.*, 1993). These tissues include brain, cerebellum, heart, skeletal muscle, lung, kidney, liver, spleen, thyroid, adrenal gland and ovary. However, the signal for *Tie* mRNA is reduced in the endothelia of neural tissues of adult rats when compared with the embryonic and neonatal neural tissue. Expression of *Tie* mRNA has also been detected in human haematopoietic progenitor cells (Batard *et al.*, 1996; Hashiyama *et al.*, 1996), in leukaemia cell lines and in some cell lines from solid tumours (Partanen *et al.*, 1992; Armstrong *et al.*, 1993).

Several studies have pointed out the importance of angiogenesis for tumour growth and progression (Folkman, 1990, 1992). Microvessel density has been shown to be an independent parameter of the severity of tumour disease and a predictor of prognosis of breast cancer patients in most but not all studies addressing this issue (Weidner *et al.*, 1991, 1992; Bosari *et al.*, 1992; Horak *et al.*, 1992; Toi *et al.*, 1993, Axelsson *et al.*, 1995). A similar correlation has also been demonstrated with other human malignancies, including head and neck squamous cell carcinoma (Gasparini *et al.*, 1993), lung adenocarcinoma (Yamazaki *et al.*, 1994), rectal carcinoma (Saclarides *et al.*, 1994), testicular germ cell tumours (Olivarez *et al.*, 1994), prostate cancer (Weidner *et al.*, 1993), tumours of the oral cavity (Williams *et al.*, 1994) and gastric carcinoma (Maeda *et al.*, 1995). Various endothelial cell specific antibodies have been used in immunohistochemistry to quantitate blood vessels within the tumours. Antigens used for this purpose include von Willebrand factor, CD31 (PECAM) and CD34. The purpose of this work was to study the *Tie* protein in angiogenesis associated with breast cancer. Our aim was also to evaluate the utility of *Tie* detection as a measure of breast tumour angiogenesis and the association of *Tie* expression with prognostic factors in breast cancer.

Correspondence: P Salvén, Department of Oncology, Helsinki University Central Hospital, Haartmaninkatu 4C, 00290 Helsinki, Finland
Received 28 September 1995; revised 2 January 1996; accepted 15 January 1996

### Materials and methods

*Tissue sources*

Freshly frozen sections of 56 tissue samples in total containing both normal and malignant tissues were retrieved from the histopathological files of the Department of Pathology, University of Helsinki. The tissues examined included 19 infiltrating ductal carcinomas, six infiltrating lobular carcinomas, two infiltrating tubular carcinomas, one ductal carcinoma *in situ*, one lobular carcinoma *in situ*, five benign fibroadenomas, one adenosis, 16 samples of normal breast tissue, two normal axillary lymph nodes and three samples of normal breast skin.

*Immunostaining*

The primary antibodies used were: mouse monoclonal anti-*Tie*, clones 10f11 and 7e8, used on sections at a concentration of 8 $\mu$g $ml^{-1}$ and mouse anti-CD31, clone HC1/6 (Novocastra Laboratories), used on sections at a concentration of 0.66 $\mu$g $ml^{-1}$. Staining with the primary antibodies was for 1 h at room temperature. Control stainings included irrelevant monoclonal antibodies of the same isotype as well as anti-*Tie* incubated overnight with a 5-fold molar excess of the *Tie* extracellular domain expressed in baculovirus (Batard *et al*., 1996).

Frozen sections (5 $\mu$m) on slides were dried at room temperature overnight. Following rehydration in phosphate-buffered saline (PBS) for 5 min the sections were overlaid with normal horse serum for 20 min before incubation with the primary antibody. Subsequent incubation for 30 min in biotinylated anti-mouse serum was followed by a 30 min incubation using reagents of the Vectastain Elite ABC kit (Vector laboratories). Peroxidase activity was developed with 3-amino-9-ethyl carbazole (Sigma) for 10 min. Finally, the sections were stained with haematoxylin for 5 min.

Following the staining procedures, all samples were examined by a trained pathologist. The highest microvessel counts were assessed according to Weidner *et al.* (1991). After the area of highest amount of stained microvessels (so-called vascular hotspots) was identified by light microscopy, individual stained microvessels were counted using a 400 × magnification field (i.e. 40 × objective lens and 10 × ocular lens). Each count was expressed as the highest number of stained microvessels identified within any high-power field (hpf).

*Statistical analysis*

Staining count distributions of different groups were analysed using Kruskal–Wallis's analysis of variance and the Mann–Whitney test.

### Results

*Tie protein in histologically normal tissue*

*Tie* protein was consistently detected in the microvessel endothelial cells of histologically normal dermis ($n$=3), axillary lymph nodes ($n$=2) and breast tissue ($n$=16) and with anti-*Tie* antibodies clones 7e8 and 10f11 (Figure 1a and b). Antibody of clone 10f11 stained breast myoepithelial cells in some of the samples, but the staining intensity was weaker than for endothelial cells. Microvessel counts per hpf were

**Figure 1** Peroxidase immunostaining of *tie* in normal and pathological tissues. MAb 7e8 staining of normal skin (**a**) and breast (**b**) as well as invasive ductal carcinoma (**c**). Staining with *tie* antigen-blocked MAb is also shown (d). Scale bar = 100 $\mu$m.

higher when staining was performed using the clone 7E8 antibody (Table I). No specific staining was observed when the sections were incubated with antigen-blocked antibody instead of the primary antibodies, or with the peroxidase-conjugated secondary antibody only.

*Tie protein in breast tumours*

Microvessel counts in fibroadenomas were not significantly higher than those found in normal breast when the anti-*Tie* antibody clone 7e8 was used (median, 10 per hpf *vs* 7 respectively, $P=0.12$). The microvessel counts were higher in fibroadenomas than in histologically normal breast tissue when anti-CD31 staining was used (median, 21 *vs* 15 respectively, $P=0.02$). *Tie* expression was also detected in vessels around intraductal cancer and lobular carcinoma *in situ* with the anti-*Tie* antibody clone 7e8.

*Tie* protein was detected in the endothelial cells of microvessels in all breast carcinoma samples studied with the clone 7e8 antibody (Figure 1c). The number of stained microvessels was greater in breast carcinomas than in normal breast tissue when staining was performed with the clone 7e8 antibody (median, 14 *vs* 7 respectively, $P=0.0002$). The highest microvessel counts were also clearly greater in breast cancer tissue than in normal breast tissue in staining for CD31 (median, 30 *vs* 15 respectively, $P<0.0001$). No specific staining was observed when the tumour sections were incubated with antigen-blocked antibody or normal serum instead of the anti-*Tie* antibodies (Figure 1d).

In some of the invasive breast carcinoma samples the anti-*Tie* antibody clone 10f11 also stained carcinoma cells, basement membranes and myoepithelial cells, whereas the clone 7e8 antibody stained vascular endothelial cells only.

The highest microvessel counts obtained by staining for *Tie* protein with the two anti-*Tie* antibodies and for CD31 were correlated with the primary tumour size (<2 cm *vs* >2 cm), presence of axillary nodal metastases (pN0 *vs* pN+), histological gradus (well- or moderately differentiated *vs* poorly differentiated), oestrogen and progesterone receptor status, Ki-67 expression (lower *vs* higher than the median, 15%), and p53 expression (negative *vs* positive) among the 27 carcinomas studied, but no significant correlations were found between these parameters and the microvessel counts.

### Discussion

Several studies have suggested that the *Tie* protein plays an important role in angiogenesis (Partanen *et al*., 1992; Sato *et al*., 1993; Korhonen *et al*., 1994, 1995). In recent studies high amounts of *Tie* mRNA and also *Tie* protein have been detected in human brain tumours in contrast to the less abundant expression of *Tie* mRNA or protein in the respective normal brain tissue control samples (Kaipainen *et al*., 1994; Hatva *et al*., 1995). These studies suggest that a significant difference exists in the expression of *Tie* when the endothelia of malignant tumours of the CNS are compared with the endothelia of normal adult brain.

Our results show *Tie* protein in the vascular endothelia of several types of normal human tissues, including normal skin and breast tissue. In a previously published work with human melanoma and normal skin samples using *in situ* hybridisation the *Tie* probe hybridised very weakly with the vascular endothelium of capillaries of normal skin, except for the endothelium of sweat gland vessels (Kaipainen *et al*., 1994). In addition, *Tie* expression appeared to be enhanced in areas of inflammatory reaction around certain skin melanomas. The differences between the results in this *in situ* hybridisation and the present immunohistochemical staining results can be due to a tissue-specific variation in the level of *Tie* expression, a difference in the sensitivity of detection techniques used or translational regulation of *Tie* expression. The fact that the 10f11 antibody cross-reacts with an antigen expressed in ductal myoepithelium and some breast carcinomas is not unexpected among monoclonal antibodies, which often detect very small epitopes. Such epitopes may resemble structures in other proteins. However, at least in brain tumours and melanomas *Tie* sequences as such are only expressed in tumour endothelia and not detected in *in situ* hybridisation of the tumour cells (Hatva, *et al*., 1995; Kaipainen, *et al*., 1994) excluding the possibility that *Tie* would be aberrantly expressed at least in a significant fraction of myoepithelial or breast carcinoma cells.

In contrast to the expression of CD31 we did not detect *Tie* expression in all microvessels of normal or malignant breast tissue. Thus the *Tie* antigen may not be expressed in all endothelial cells. A more likely explanation is, however, that the level of *Tie* expression is so low that it does not allow immunohistochemical detection. One parameter affecting the staining intensity is obviously the thickness of the histological sections used for staining and another concerns the possible masking of the *Tie* epitope recognised by the antibodies in tissue sections.

Tumour angiogenesis is essential for tumour growth and metastasis and intratumoral microvessel density correlates with prognosis in breast carcinoma and also in other human tumours. In this study the difference in the *Tie*-positive microvessel counts between the groups of invasive breast carcinomas and normal breast tissue samples was statistically significant (Mann–Whitney test, $P=0.0002$). The difference between these groups was similar to the difference observed when using the anti-CD31 antibody. This result suggests that *Tie* might have significance as an indicator of tumour angiogenesis and as a prognostic marker for breast cancer patients. Although the microcapillary network of breast carcinomas is known to be unevenly distributed, our results do not suggest that *Tie* would be up-regulated in breast carcinomas. Whether *Tie* is enhanced in tumour vessels outside the central nervous system needs further study using more quantitative methods of analysis. Thus we cannot yet exclude scenarios of tumour treatment based on possible differential *Tie* expression in normal and tumour endothelia. However, the *Tie* receptor may appear on the luminal surface of endothelial cells and circulating *Tie* antigen may be present in the blood. Such findings could preclude the use of anti-*Tie* antibodies for intravascular injection because of the possible

**Table I**

| *Antibody* | *Normal breast* (n=*16*) | *Fibroadenoma* (n=*5*) | *Breast cancer* (n=*27*) | P *Kruskal–Wallis test* |
|---|---|---|---|---|
| 7e8 | | | | |
| Range | 3–15 | 5–18 | 3–27 | |
| Median | 7 | 10 | 14 | <0.0006 |
| CD31 | | | | |
| Range | 8–30 | 18–28 | 18–40 | |
| Median | 15 | 21 | 30 | <0.0001 |

Statistical analysis of microvessel counts using the anti-*tie* antibody clone 7e8 and the anti-CD31 antibody in normal breast tissue, fiboradenoma and invasive breast cancer formation of immunocomplexes with adverse side-effects. However, it remains possible that inhibition of *Tie* function by, e.g. interference with *Tie*-ligand interaction or *Tie*-specific signal transduction, could prevent tumour angiogenesis.

**Acknowledgements**

We thank Eola Kukk and Drs Juha Partanen and Riitta Alitalo for collaboration in the generation of anti-*Tie* monoclonal antibodies. The skilful technical assistance of Ms Päivi Heino, Mrs Päivi Laurila, Ms Mari Helanterä, Mrs Tuula Lindholm is gratefully acknowledged. We thank Dr Vijay Kumar for checking the language. This study was supported by The Finnish Academy, The Ida Montin Foundation, The Finnish Cancer Organizations, The Sigrid Juselius Foundation and the State Technology Development Centre. We thank Dr Vijay Kumar for chekcing the language

### References

ARMSTRONG E, KORHONEN J, SILVENNOINEN O, CLEVELAND JL, LIEBERMAN MA AND ALITALO R. (1993). Expression of Tie receptor tyrosine kinase in leukemia cell lines. *Leukemia*, **7**, 1585–1591.

AXELSSON K, LJUNG B-M, MOORE D, THOR A, CHEW K, EDGERTON S, SMITH H AND MAYALL B. (1995). Tumor angiogenesis as a prognostic assay for invasive ductal breast carcinoma. *J. Natl Cancer Inst.*, **13**, 997–1008.

BATARD P, SANSILVESTRI P, SCHNEINECKER C, KNAPP W, DEBILI N, VAINCHENKER W, BÜHRING H-J, MONIER M-N, KUKK E, PARTANEN J, MATIKAINEN M-T, ALITALO R, HATZFELD J AND ALITALO K. (1996). The Tie receptor tyrosine kinase is expressed by human hematopoietic progenitor cells and by a subset of megakaryocytic cells. *Blood*, **86**, 1729–1735.

BOSARI S, LEE A, DELELLIS R, WILEY B, HEATLEY G AND SILVERMAN M. (1992). Microvessel quantitation and prognosis in invasive breast carcinoma. *Hum. Pathol.*, **23**, 755–761.

DUMONT DJ, GRADWOHL GJ, FONG G-H, AUERBACH R AND BREITMAN ML. (1993). The endothelial-specific receptor tyrosine kinase, tek, is a member of a new subfamily of receptors. *Oncogene*, **8**, 1293–1301.

FOLKMAN J. (1990). What is the evidence that tumors are angiogenesis dependent? *J. Natl Cancer Inst.*, **82**, 4–6.

FOLKMAN J. (1992). The role of angiogenesis in tumor growth. *Semin. Cancer Biol.*, **3**, 65–71.

GASPARINI G, WEIDNER N, MALUTA S, POZZA F, BORACCHI P, MEZZETTI M, TESTOLIN A AND BEVILACQUA P. (1993). Intratumoral microvessel density abd p53 protein: correloation with metastasis in head-and-neck squamous-cell carcinoma. *Int. J. Cancer*, **55**, 739–744.

HASHIYAMA M, IWAMA A, OSHIRO K, KUROZUMI K, YASUNGA K, SHIMIZU Y, MASUHO Y, MATSUDA I, YAMAGUCHI N AND SUDA T. (1996). Predominant expression of a receptor tyrosine kinase, Tie, in hematopoietic stem cells and B cells. *Blood*, **87**, 93–101.

HATVA E, KAIPAINEN A, JÄÄSKELÄINEN J, HALTIA M AND ALITALO K. (1995) Endothelial cell-specific receptor tyrosine kinases and growth factors in human gliomas and meningiomas. *Am. J. Pathol.* **146**, 368–378.

HORAK E, LEEK R, KLENK N, LEJEUNE S, SMITH K, STUART N, GREENALL M, STEPNIEWSKA K AND HARRIS A. (1992). Angiogenesis, assessed lately endothelial cell adhesion molecule antibodies, as indicator of node metastasis and survival in breast cancer. *Lancet*, **340**, 1120–1124.

KAIPAINEN A, VLAYKOVA T, HATVA E, BÖHLING T, JEKUNEN A, PYRHÖNEN S AND ALITALO K. (1994). Enhanced expression of the Tie receptor tyrosine kinase mRNA in the vascular endothelium of metastatic melanomas. *Cancer Res.*, **54**, 6571–6577.

KORHONEN J, PARTANEN J, ARMSTRONG E, VAAHTOKARI A, ELENIUS K, JAKLANEN M AND ALITALO K. (1992) Enhanced expression of the Tie receptor tyrosine kinase in endothelial cells during neovascularisation. *Blood*, **15**, 2548–2555.

KORHONEN J, POLVI A, PARTANEN J AND ALITALO K. (1994). The mouse Tie receptor tyrosine kinase gene: expression during embryonic angiogenesis. *Oncogene*, **9**, 395–403.

KORHONEN J, LAHTINEN I, HALMEKYTÖ M, ALHONEN L, JÄNNE J, DUMONT D AND ALITALO K. (1995). Endothelial-specific gene expression directed by the Tie gene promoter in vivo. *Blood*, **5**, 1828–1835.

MAEDA K, CHUNG Y, TAKATSUKA S, OGAWA Y, SAWADA T, YAMASHITA Y, ONODA N, KATO Y, NITTA A, ARIMOTO Y, KONDO Y AND SOWA M. (1995). Tumor angiogenesis as a pedictor of recurrence in gastric carcinoma. *J. Clin. Oncol.*, **13**, 477–481.

MAISONPIERRE PC, GOLDFARB M, YANCOPOULOS GD AND GAO G. (1993). Distinct rat genes with related profiles of expression define Tie receptor tyrosine kinase family. *Oncogene*, **8**, 1631–1637.

OLIVAREZ D, ULBRIGHT T, DERIESE W, FOSTER R, REISTER T, EINHORN L AND SLEDGE G. (1994). Neovascularization in clinical stage A testicular germcell tumor: prediction of metastatic disease. *Cancer Res.*, **54**, 2800–2802.

PARTANEN J, ARMSTRONG E, MÄKELÄ T, KORHONEN J, SANDBERG M, RENKONEN R, KNUUTILA S, HUEBNER K AND ALITALO K. (1992). A novel endothelial cell surface receptor tyrosine kinase with extracellular epidermal growth factor homology domains. *Mol. Cell. Biol.*, **12**, 1698–1707.

PURI M ROSSANT J, ALITALO K, BERNSTEIN A AND PARTANEN J. (1995). The receptor tyrosine kinase Tie is required for the integrity and survival of vascular endothelial cells. *EMBO J.*, **23**, 5884–5891.

SACLARIDES T, SPEZIALE N, DRAB E, SZELUGA D AND RUBIN D. (1994). Tumor angiogenesis and rectal carcinoma. *Dis. Colon. Rectum.*, **37**, 921–926.

SATO T, QIN Y, KOZAK CA AND AUDUS K. (1993). Tie-1 and Tie-2 define another class of putative receptor tyrosine kinase genes expressed in early embryonic vascular system. *Proc. Natl Acad. Sci. USA*, **90**, 9355–9358.

SATO T, TOZAWA Y, DEUTSCH U, WOLBURG-BCHHOLZ K, FUJIWARA Y, GENDRON-MAGUIRE M, GRIDLEY T, WOLBURG H, RISAU W AND QIN Y. (1995). Distinct roles of the receptor tyrosine kinases Tie-1 and Tie-2 in blood vessel formation. *Nature*, **376**, 70–74.

TOI M, KASHITANI J AND TOMINAGA T. (1993). Tumor angiogenesis is an independent prognostic indicator in primary breast carcinoma. *Int. J. Cancer*, **55**, 371–374.

VAN DER GEER P, HUNTER T AND LINDBERG RA. (1994). Receptor protein-tyrosine kinases and their signal transduction pathways. *Annu. Rev. Cell Biol.*, **10**, 251–337.

WEIDNER N, CARROLL P, FLAX J, BLUMFELD W AND FOLKMAN J. (1993). Tumor angiogenesis correlates with metastasis in invasive prostate carcinoma. *Am. J. Pathol.*, **143**, 401–409.

WEIDNER N, FOLKMAN J, POZZA F, BEVILACQUA P, ALLRED E, MOORE D, MELI S AND GASPARINI G. (1992). Tumor angiogensis: a new significant and independent prognostic indicator in early-stage breast carcinoma. *J. Natl Cancer Inst.*, **82**, 1875–1887.

WEIDNER N, SEMPLE J, WELCH W AND FOLKMAN J. (1991). Tumor angiogenesis and metastasis–correlation in invansive breast carcinoma. *N. Engl. J. Med.*, **324**, 1–8.

WILLIAMS J, CARLSON G, COHEN C, DEROSE P, HUNTER S AND JURCIEWICZ M. (1994). Tumor angiogenesis as a prognostic factor in oral cavity tumors. *Am. J. Surg.*, **168**, 373–380.

YAMAZAKI K, ABE S, TAKEKAWA H, SUKOH N, WATENABE N, OGURA S, NAKAJIMA I, ISOBE H, INOUE K AND KAWAKAMI Y. (1994). Tumor angiogenesis in lung adenocarcinoma. *Cancer*, **74**, 2245–2250.

ZIEGLER SF, BIRD TA, SCHNERINGER JA, SCHOOLEY KA AND BAUM PR. (1993). Molecular cloning and characterization of a novel receptor protein tyrosine kinase from human placenta. *Oncogene*, **8**, 663–670.